# Supplementary material for: Transcriptomic and phylogenetic analysis of a bacterial cell cycle reveals strong associations between gene co-expression and evolution
Source: BMC Genomics. 2013 Jul 5;14:450. doi: 10.1186/1471-2164-14-450 (PMC3829707; doi:10.1186/1471-2164-14-450)
Supplement: Additional file 9: Figure S2 — Expression profiles of all identified CCR genes. [file 1471-2164-14-450-S9.zip › FigureS2/CCNA_03589.pdf]

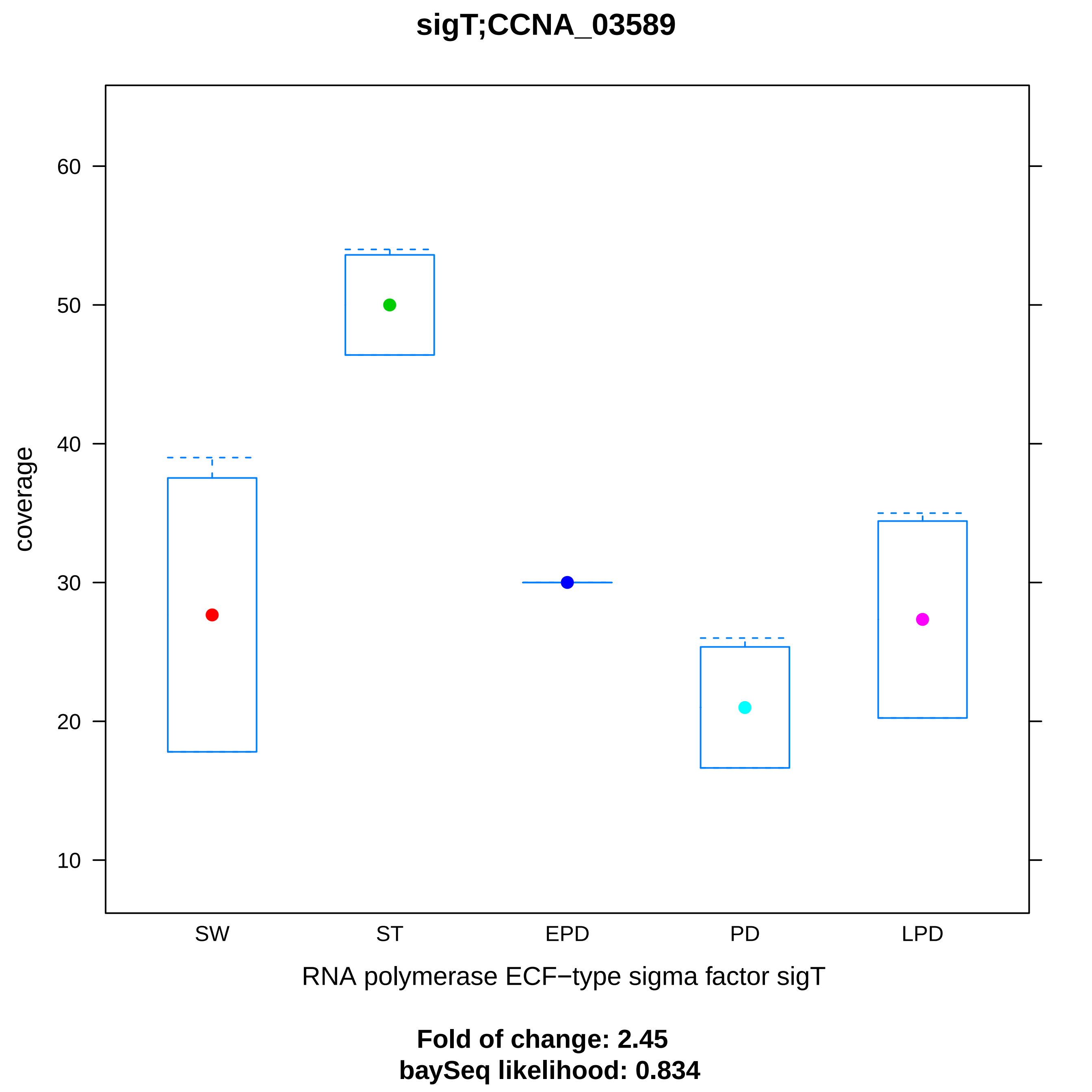

sigT;CCNA_03589
coverage
60
50
40
30
20
10
SW
ST
EPD
PD
LPD
RNA polymerase ECF−type sigma factor sigT
Fold of change: 2.45
baySeq likelihood: 0.834
